# The Focal Attention Window Size Explains Letter Substitution Errors in Reading

**DOI:** 10.3390/brainsci11020247

**Published:** 2021-02-16

**Authors:** Roberta Daini, Silvia Primativo, Andrea Albonico, Laura Veronelli, Manuela Malaspina, Massimo Corbo, Marialuisa Martelli, Lisa S. Arduino

**Affiliations:** 1 Department of Psychology, University of Milano-Bicocca, 20126 Milan, Italy; andrea.albonico@gmail.com (A.A.); manu.malaspina@gmail.com (M.M.); 2 COMiB—Optics and Optometry Research Center, Università degli studi di Milano-Bicocca & NeuroMI—Milan Center for Neuroscience, 20126 Milan, Italy; 3 Department of Human Sciences, LUMSA University, 00193 Rome, Italy; s.primativo@lumsa.it (S.P.); l.arduino@lumsa.it (L.S.A.); 4 Departments of Ophthalmology and Visual Sciences, University of British Columbia, Vancouver, BC V5Z 3N9, Canada; 5 Department of Neurorehabilitation Sciences, Casa di Cura del Policlinico, 20144 Milan, Italy; l.veronelli@ccppdezza.it (L.V.); m.corbo@ccppdezza.it (M.C.); 6 Department of Psychology, Sapienza University of Rome, 00185 Rome, Italy; marialuisa.martelli@uniroma1.it; 7 Neuropsychology Unit, IRCCS Fondazione Santa Lucia, 00179 Rome, Italy

**Keywords:** neglect dyslexia, crowding phenomenon, substitution errors, single word reading, focal attention

## Abstract

Acquired Neglect Dyslexia is often associated with right-hemisphere brain damage and is mainly characterized by omissions and substitutions in reading single words. Martelli et al. proposed in 2011 that these two types of error are due to different mechanisms. Omissions should depend on neglect plus an oculomotor deficit, whilst substitutions on the difficulty with which the letters are perceptually segregated from each other (i.e., crowding phenomenon). In this study, we hypothesized that a deficit of focal attention could determine a pathological crowding effect, leading to imprecise letter identification and consequently substitution errors. In Experiment 1, three brain-damaged patients, suffering from peripheral dyslexia, mainly characterized by substitutions, underwent an assessment of error distribution in reading pseudowords and a T detection task as a function of cue size and timing, in order to measure focal attention. Each patient, when compared to a control group, showed a deficit in adjusting the attentional focus. In Experiment 2, a group of 17 right-brain-damaged patients were asked to perform the focal attention task and to read single words and pseudowords as a function of inter-letter spacing. The results allowed us to confirm a more general association between substitution-type reading errors and the performance in the focal attention task.

## 1. Introduction

Reading is complex behaviour that requires the interaction of many spared and fully developed cognitive capacities, such as attention, perception, and lexical access. The consequence is that the atypical development of any of the functions involved, as much as a brain lesion compromising the neural substrate, may result in a reading deficit (i.e., dyslexia) with diverse phenotypes characterized by low- and/or high-level error types. The study of acquired central and peripheral dyslexias in adults could be very informative in isolating some of the compromised mechanisms.

In the present study, we have focused on one of these specific acquired reading disorders, namely Neglect Dyslexia (ND), which can shed some light on attentional and perceptual components in reading. ND is characterized by reading errors in the contralesional side of sentences and texts, but also single words, irrespective of presentation times. Errors in single-word reading are considered to be markers for ND [1]. It is often associated (40%, according to Lee et al. [2]) with other manifestations of Unilateral Spatial Neglect (USN). Patients affected by such a neuropsychological syndrome fail to explore the side of space contralateral to the lesion and, within a given coordinate system (e.g., egocentric, object-based), to report stimuli presented in that portion of space (e.g., [3]). USN includes a set of deficits that do not concern the initial levels of information processing, but those of higher order, such as visual consciousness (3). Any primary visual disturbances (i.e., visual field defect, as lateral homonymous hemianopia) concern retinotopic coordinates, and, although frequently associated with NSU, have shown a double dissociation from USN by electrophysiological, behavioural, and anatomical evidence (e.g., [4]).

ND is classified as a “peripheral” dyslexia, namely, a disorder affecting the initial pre-lexical stages of reading [5,6]. These early stages involve a visual feature analysis of the letter string’s individual components, which activate the appropriate letter representations in a system of abstract letter units (see [7,8]), and these stages, in turn, are affected by attention and eye-movements [6]. The process of letter identification precedes reading proper and is common to both word and pseudoword reading aloud. “Peripheral” dyslexias are distinct from “central” dyslexias [5,6], which are characterized by damage to one or more of the components specific to the reading routines (i.e., lexical and sublexical components according to the dual route cascaded model of visual word recognition by Coltheart et al. [9]. Within such a framework, ND may be distinguished from the other two main types of peripheral dyslexias (pure alexia and attentional dyslexia; e.g., [5,6]) in that reading errors mostly involve the contralesional side of space, most of the time the left side of the letter string. In order to clarify from the start the types of reading deficit that we considered in the paper, we firstly described their features. Central and peripheral dyslexia differ from each other not only in terms of mechanisms involved but also in terms of errors made. Regarding single word reading, errors are visual and are not influenced by phonology (i.e., do not respect the grapheme/phoneme organization of words) or semantics. The most common errors in ND are: (i) omissions [e.g., the word “candela” (candle) read as a nonword “dela”] and (ii) substitutions [e.g., the word “lista” (list) read as pista “track”]. The relative proportion of these error types varies across patients. A predominance of substitution errors has been reported for some patients [1,10,11], even though in the vast majority of cases omission errors predominate [8,12,13,14]. For many years, the classification of neglect dyslexia errors has been made using the neglect-point measure of Ellis et al. [1]. This measure defines neglect errors as “errors in which target and error words are identical to the right of an identifiable neglect point in each word, but share no letters in common to the left of the neglect point” (p. 445). This conservative measure ensures that errors are likely coming from neglect, rather than from any other pathological source. With this in mind, in single word reading, patients have been described in terms of their prevalence to produce one type of error (substitutions and omissions of contralesional letters) and their sensitivity to the lexical status of the target producing less errors in reading words than pseudowords and high-frequency words than low frequency words (e.g., [15,16]; for a review see Vallar et al. [17]). By putting these two aspects together (the patients’ difference in their prevalence of substitutions vs. omissions and their different sensitivity to the lexical status of the target), an interpretative model has been proposed. MORSEL [18,19] is a computational model that integrates a word recognition system similar to McClelland and Rumelhart’s Interactive Activation Model [20] and an attentional mechanism, with high-level knowledge interacting with perceptual processing. In the damaged version of the model, developed to simulate and interpret the performance of patients with left unilateral spatial neglect in reading, there is less probability for left-sided information to be processed. Following MORSEL [19], neglect dyslexia could be considered as a consequence of a single functional impairment in a single attentional mechanism, which can be disrupted along a continuum of severity [18,19]. According to this view, a milder deficit would account for substitution errors, whereas one that is more severe would produce omission errors [15].

However, in describing RCG, a right-brain-damaged patient who manifested a spatial reading disorder that was characterized mostly by left-sided substitutions and without any other sign of USN, Arduino, Daini, and Silveri [21] suggested that substitution errors might not be directly related to the unilateral spatial disorder. Furthermore, the reading performance of patient RCG was sensitive to spacing. Indeed, the total number of reading errors decreased significantly when the inter-letter spacing was increased, and despite that, the letter string occupied a larger portion of the neglected space after this manipulation. According to the authors, this finding suggested that substitutions may depend on a different mechanism from that responsible for omissions, and that perceptual integration may play a crucial role in determining substitution errors in brain-damaged patients [21].

Subsequently, Martelli et al. [22] proposed a dual model and hypothesized that substitution and omission errors produced by neglect dyslexia patients in reading could in fact be due to independent mechanisms. The first would consist of a visuospatial mechanism responsible for omissions in both ND and USN (similar to left-sided errors in cancellation tasks), whereas the second mechanism, causing a predominance of substitutions, would be perceptual in nature and independent of USN. Supporting the hypothesis that the same visuospatial mechanism is responsible for omission errors in both ND and USN, Martelli et al. [22] found that omissions, but not substitutions, tend to be related to the severity of neglect. Such a relationship has been further confirmed by Weinzierl et al. [23]. The authors compared reading errors in USN patients and normal controls, with a reduced word presentation time in normal controls in order to elicit reading errors. The authors found a linear left-right gradient of errors in USN patients, whatever the error type, whereas errors in controls were more equally distributed on the different letter locations in the word. They also found that omission errors, and not substitutions, were pathognomonic of ND.

Omissions in single word and pseudoword reading (i.e., ND) are associated to USN but do not overlap. Less than half of the NSU patients show ND [2], and patients with severe neglect can be spared when reading central view letter strings. The actual models do not explain why only some patients with USN show the co-occurrence of ND. A study by Primativo et al. [24] demonstrated that neglect patients with ND (all characterized by left lateralized omission errors) showed an abnormal eye movement pattern in both a reading task and a non-verbal saccadic task compared to neglect patients without ND and to control right-brain-damaged patients without USN. In particular, ND patients made a great amount of inaccurate fixations: specifically, their fixations did not fall on the stimulus but were distributed in different positions on the screen. Thus, Primativo et al. [24] concluded that the omission errors in ND are the phenotypic expression of the co-occurrence of the exploratory deficit (USN) and an altered oculomotor pattern, which prevents the automatic execution of the fine eye movements required for reading. Daini et al. [25] and Primativo et al. [26] confirmed the association between omission errors in reading and an abnormal eye-movement pattern in USN patients. Nonetheless, substitution errors are not associated with NSU [22] nor with eye movement alterations [25]. Martelli et al. [22] proposed that substitution errors would depend on crowding, that is, a perceptual integration process that limits letter identification [27,28]. In crowding, the signal to be identified does not disappear, but remains visible, even if unrecognizable [27]. Accordingly, a substitution error is a visual error, equivalent to a misidentification, in which the letter signal is detected, but the identity of the letter is confused [22]. Crowding characterizes the normal periphery, and different studies have shown that it could be diminished while restoring letter recognition by increasing spacing [27,28]. Therefore, the fact that (i) patients with ND make substitution errors similar to those made by normal readers when reading letter strings in the periphery of the visual field, and that (ii) increasing the inter-letter spacing only reduces substitution errors, whereas it increases omissions [22], suggests that crowding could be an ideal candidate for the explanation of substitution errors.

Further evidence for the independence of the mechanisms underlying omission and substitution errors comes from a study investigating the different effects of an optokinetic stimulation session (OKS) performed with two patients showing a predominance of one or the other type of error [25]. OKS is a technique that facilitates the displacement of the oculomotor exploration towards the neglected side of the space, thus restoring oculomotor scanning [29]. According to the hypothesis that only omission errors are due to abnormal oculomotor behaviour in both reading and non-reading tasks, Daini et al. [25] found that the OKS significantly reduced the number of omissions but not that of substitutions.

A similarly well understood mechanism underlying the substitution errors has not yet been provided in the literature, and this represents the aim of the present study. More specifically, we have sought to examine the possible role of crowding, as proposed by Martelli et al. [22]. Indeed, since some studies argue that crowding reflects the limitation of the spatial resolution of attention [30,31,32], and that focal attention might play a critical role in foveal vision [33], and specifically in foveal crowding [34], we have investigated the possibility that a deficit in the focal component of the spatial attentional could play an important role in determining substitution errors in reading by enhancing crowding.

In Experiment 1, we selected as single cases those brain-damaged patients who suffered from peripheral dyslexia which was mainly characterized by substitutions. They were assessed for neglect and neglect dyslexia and underwent a T-detection task as a function of cue characteristics and timing. This test allows us to control and adjust the size of the attentional focus to be measured according to the cue characteristics and under different temporal constraints [33]. We expected that the patients presenting substitution errors in reading, compared to the control group, would show a deficit in the ability to adjust the attentional focus.

A second experiment was carried out following the opposite logic. The participants were first evaluated on the basis of their focal attention performance, and then their single-word reading performance was assessed as a function of inter-letter spacing. This strategy allowed us to verify the relationship between substitution-type reading errors and a low performance in the focal attention task (i.e., a lower or absent cue-size effect).

We expected to find that the patients suffering from a focal attention deficit were those with a prevalence of substitution errors in reading words and pseudowords.

Finally, if they showed a reduction of substitution errors with increasing spacing, then we could confirm the role of the crowding phenomenon in the relationship between focal attention and substitution errors in reading.

## 2. Experiment 1

In order to verify the hypothesized relationship between substitution errors and impaired focal attention, we ran a pilot experiment by selecting patients with neglect dyslexia who showed a prevalence of substitution errors. Therefore, we compared the performance of each of our patients with a control group in a paradigm of attentional focus. Indeed, if an impaired control of the size of the attentional window increased the crowding effect in these patients, preventing the letters from being isolated and correctly identified, this should result in a misperception of the individual letters (i.e., substitutions) and non-lateralized errors.

### 2.1. Participants

#### 2.1.1. Control Participants

A total of 12 healthy participants (2M, mean age 78.0 ± 6.2 years, age range 66–85, mean education 9.7 ± 5.1 years, education range 4–17) took part in the study and served as the control group. They were all right-handed and had no history of previous neurological and psychiatric disorders.

#### 2.1.2. Brain-Damaged Patients with Reading Difficulties

Patients were recruited by screening a wider population at the Casa di Cura Privata del Policlinico, Milan. Three patients who fitted the criteria for inclusion (i.e., neglect dyslexia and a prevalence of substitution errors) and who volunteered to participate were assessed at the Department of Psychology at the Università degli studi di Milano—Bicocca, over a period of one year. The patients were all right-handed and had no history of previous neurological and psychiatric disorders. The demographic and neurological characteristics of the patients are summarized in Table 1.

The study was approved by the local Ethical Committee of the University of Milano-Bicocca, and informed consent was obtained from all participants, in accordance with the guidelines of the Declaration of Helsinki.

#### 2.1.3. Neuropsychological Assessment

In order to assess the presence and severity of unilateral spatial neglect and neglect dyslexia, the patients underwent different tests (see Table 2): Bells cancellation [35], line bisection [36], single-word and single-pseudoword reading test [37]. The patients were considered as having ND if 50% or more of their reading errors were classified as neglect errors in both word and pseudoword reading tasks. Neglect errors refer to all misread items with left-sided errors, according to the Ellis et al. [1] criterion, whereas omissions and substitutions refer, respectively, to all neglect errors in which the length of the item produced was shorter than, or the same as, that of the target.

The results from the neuropsychological assessment demonstrated that RE and AS did not show USN, whereas CG exhibited a shift of the manual bisection to the right side, but no USN in the Bells cancellation task. Moreover, CG presented a very mild aphasic impairment. Comprehension skills were adequate. The language comprehension and production of RE and AS were adequate.

All three patients showed reading difficulties that can be classified as ND, and all were mostly characterized by substitutions (substitution-type ND).

### 2.2. Materials and Methods

**Letter by letter analysis of reading errors.** After the neuropsychological assessment we needed to describe and characterize the type of reading errors made by the three patients better, according to the letter position analysis used by Martelli et al. [22]. Indeed, the Ellis et al. [1] criterion that we used for the diagnosis of ND only takes into account the errors from the left side of the neglected point, often dismissing those occurring on the right part of the stimulus. In contrast, crowding predicts that errors would be distributed both on the right and left sides of the stimulus, with a decrease in the number for the more external and foveal letters [22]. Thus, the measurement of reading errors over the entire stimulus would allow us to obtain a more complete assessment of the omission and substitution errors made by the patients and to obtain a more detailed description of their performance. Finally, to avoid the effect of inaccurate fixations, we used a central presentation controlled by means of an eye tracker that the stimulus exploration started from its objective centre.

A list of 40 pseudowords was created by interchanging the syllables of existing words [38] in random positions, in order to preserve pronunciation and minimize word similarity. Pseudowords varied in length, from five to eight letters (10 stimuli for each length). The stimuli were printed in the capital Courier New font, which is characterized by consistent letter spacing, and character size was kept constant (40 pt) and subtended 1.0°. Pseudowords were written in black and presented on a white background.

The participants were seated in front of a 17-inch cathode-ray tube (CRT) monitor. A chin and forehead rest stabilized their head position and kept the viewing distance constant at 57 cm. The eye movements of the participants were monitored by an SR Research EyeLink 1000 eye-tracker controlled by SR Research Experiment Builder software (SR Research Ltd., Canada), in order to control fixation accuracy.

In each trial, the patients were shown two small square fixation points, vertically displaced 1.5° apart in the centre of the screen. These fixation marks remained on the screen for the entire experimental session. Stimulus onset was triggered when the patient steadily fixated on the centre of the space between the two square marks for at least 50 ms. This procedure ensured that the first fixation landed on the centre of the pseudowords. Each stimulus was presented at the centre of the screen between the fixation marks (i.e., the central letter of each stimulus was vertically aligned to the fixation marks) and remained on the screen until the onset of the patient’s response. There was no time constraint for responding, and the participants were asked to read each stimulus aloud as accurately as possible. Pseudowords appeared in a randomized order across participants. Responses were digitally recorded, and errors were later scored after listening to the recorded track.

**Experiment of attentional focus.** The aim of this experimental session was to assess the participants’ abilities in controlling the attentional focus and in adjusting it according to the task’s demands. This was accomplished by using a cueing task based on the paradigm described by Maringelli and Umiltà [39] and implemented by Albonico et al. [33].

The participants were seated in front of a computer monitor (27-inch, 600 × 340 mm); a chin and forehead rest stabilized their head position and kept the viewing distance constant at 57 cm. The recording of stimulus presentation, timing, and response was carried out using the Experiment Builder software (SR Research Ltd., Mississauga, ON, Canada).

The target stimulus consisted of a capital letter T (font Sloan, colour black) of 1° × 1° of visual angle oriented upright, whereas the cue could be represented by a red dot (0.4° diameter), a small black square (1.2° × 1.2°, 0.1° thickness), a large black square (15° × 15°, 0.1° thickness), or by no cue. All the stimuli, either cue or target, were always presented at the centre of the screen.

Each trial started with a blank screen followed, 1000 ms after, by one of the possible cues (i.e., a large or a small square, or a dot). The cue, when presented, remained on the screen until the appearance of the target, whereas the target stimulus appeared after a stimulus onset asynchrony (SOA) of 100 or 500 ms. The target stimulus remained on the screen until a response was given (or for a maximum of 2000 ms), after which the target disappeared and the next trial started. A response to the target stimulus was made by pressing the spacebar on the computer keyboard. A baseline condition consisting of the absence of any cues was also included, and in 20% of the trials (i.e., catch trials) no target stimulus was presented.

The participants were instructed to fixate on the centre of the screen, and to press the spacebar as quickly as possible in response to the target stimulus. They were also instructed to refrain from responding to the appearance of a cue and to catch trials. The participants completed a total of 160 trials. SOA conditions and cue types were presented randomly. At the beginning of the experiment, a practice session consisting of 25 trials was run in order to allow the participants to familiarize themselves with the task and to practise with response modality.

### 2.3. Results

**Letter by letter analysis of reading errors.** Letter omission and substitution errors for each stimulus were measured by using a letter-based approach, and reading errors were then modelled as a function of letter position for omissions and substitutions separately. Following Martelli et al. [22] and Daini et al. [25], the proportion of omission errors produced by the patients, being determined by the USN deficit, was fitted by a three-parameter exponential decay model, using the following equation:

$$P\left(omission\right)=a+be^{\left(-cx\right)}$$

where a is the offset, b is the amplitude, and c is the rate of change. In contrast, the proportion of substitution errors was fitted by the sum of two Gaussian distributions, according to the following equation:

$$P\left(substitution\right)=a+be\;(-{\left(x-c\right)}^{2}/d^{2})$$

where a is the offset, b is the area under the curve, c is the centre of the distribution, and d is the width. Indeed, according to crowding, the letters falling around the fixation point and the external letters (that only have one flanker nearby) should be recognizable, whereas letters in intermediate positions should be misidentified because of crowding [3]. For this reason, a two-Gaussian distribution model would be the best to describe the substitution data, with picks on the left and right side of the centrally-fixated string. Fitting was conducted with the Matlab Curve Fitting Tool. The distribution of omission and substitution errors was fitted according to the equations reported above and the parameters a, b, and c (for omission errors) and a, b, c and d (for substitution errors) were estimated for each participant using a maximum-likelhood criterion. The goodness of fit of the models were evaluated with the R-square.

**Figure 1:**
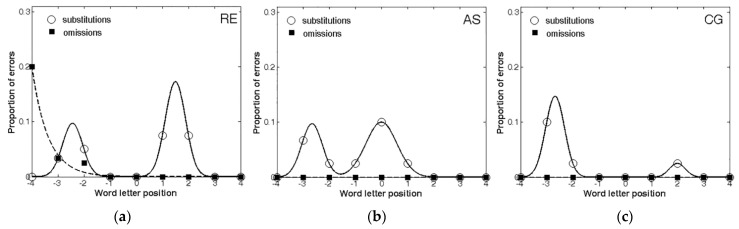
The distribution of omission and substitution errors made by the three patients: (**a**) RE; (**b**) AS; (**c**) CG. 0 corresponds to the central letter in the string, positive values to the letters on the right and negative values to the letters on the left.

Figure 1 shows the proportion of omission and substitution reading errors made by the patients as a function of letter position. The controls showed no errors or only a single error and were not analysed further.

First, all three patients made a larger number of substitutions compared to omissions, further confirming that the ND of those patients was mostly characterized by substitutions. Second, when making omissions, these errors were lateralized to the left-side of the stimulus, as predicted by USN. In contrast, substitutions were more equally distributed across the entire stimulus. The analysis of the error distribution in the three patients also confirmed, as expected, that substitutions and omissions have different shapes. Indeed, in the case of omissions, the exponential decay captured a large proportion of variance for RE (R^2^ = 0.99), whereas AS and CG did not make any omission errors at all. Instead, substitution errors showed a substantially different pattern, being well described by the bimodal distribution for RE (R^2^ = 1.00), AS (R^2^ = 0.99), and CG (R^2^ = 1.00).

Therefore, the results showed that all three patients included in the study were characterized by a substitution-type reading deficit. Furthermore, these data are consistent with previous findings, showing that patients characterized by a majority of substitutions generally produce fewer and more distributed errors, whereas omission errors tend to be left-lateralized [22,25].

**Experiment on attentional focus.** Reaction times (RTs) were adopted as the dependent measure (Table 3). Trials with false alarms (i.e., responses to catch trials and atypical RT outliers (employing as a criterion 2.5 SDs above or below the mean within each participant)) were discarded and not analysed. Responses to catch trials were rare for both patients and controls (less than 1%) and were not subsequently analysed.

Controls’ data were analysed via a two-way repeated-measures ANOVA, and the effects of interest were those associated with the experimental manipulations—that is, Cue (big square, small square, dot, and absence of a cue), SOA (100 and 500 ms), and their mutual interactions. The effect size in the ANOVA was also measured by computing the Eta Squared (η2). The cue-size effect was also computed in order to obtain an amodal quantification of the advantage of narrowing the focal component. To this end, we computed the difference in response times between the large and small square conditions for each participant, for each SOA condition. Afterwards, the mean cue-size effect for each condition was tested with a one-sample t-test against the hypothesis that they were not significantly different from zero. Finally, the cue-size effects for the patients were compared to those of the controls, using Crawford statistics [40,41].

The ANOVA for the control participants proved significant effects for both the main factors of SOA (F(1,11) = 13.91, *p* = 0.003, η^2^ = 0.169) and Cue (F(3,33) = 7.05, *p* = 0.001, η^2^ = 0.168), whereas their mutual interaction was not significant (F(3,33) = 0.756, *p* = 0.527, η^2^ = 0.017). RTs were faster for the 500 ms SOA condition (527 ms) compared to that of 100 ms (567 ms), and when the cue consisted of the small square. A planned comparison on the main effect of Cue showed that the small square condition was significantly better than the dot condition (520 and 571 ms, respectively; F(1,11) = 12.38, *p* = 0.005). Furthermore, the data on the cue-size effect highlighted the fact that the advantage of the small square was significant only when the SOA was 100 ms (44 ms; t(11) = 2.55, *p* = 0.027), whereas the effect of the mean cue-size only approached significance in the 500 ms SOA condition (37 ms; *t*(11) = 2.11, *p* = 0.059). The difference between the two SOA conditions was not significant (*t*(11) = 0.346, *p* = 0.736).

The RT analyses showed that the patients were not significantly slower than the controls (RE: 505 ms, *t*(11) = −0.325, *p* = 0.751; AS: 490 ms, *t*(11) = −0.442, *p* = 0.667; CG: 547 ms, *t*(11) = 0.000, *p* = 1.00), and that, again, the RTs of the patients were not significantly influenced by the SOA (RE: *t*(11) = 0.024, *p* = 0.981; AS: *t*(11) = 1.751, *p* = 0.108; CG: *t*(11) = 1.798, *p* = 0.0667). Table 3 lists the mean RTs of each patient and the controls.

**Figure 2:**
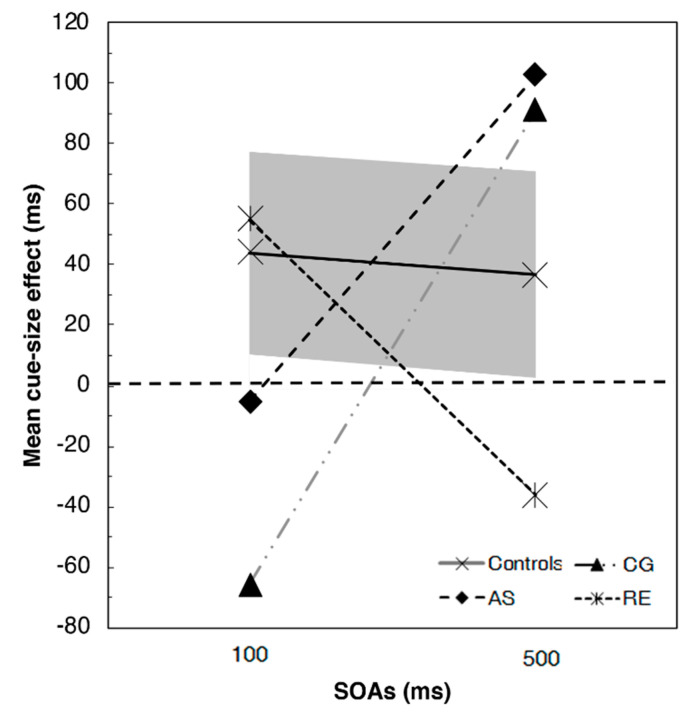
Results of the T detection task for the three patients and the control groups (the grey solid area represents the 95% confidence interval). Positive values indicate the presence of a cue size effect (i.e., faster reaction times for the small square compared to the big square) whereas negative values indicate the absence of such effect (i.e., faster reaction times for the big square compared to the small square).

The comparisons of the cue-size effect (Figure 2) confirmed that patients RE, AS, and CG had some difficulties in controlling and adjusting the size of the attentional focus. Indeed, both AS and CG showed an inversion of the cue-size effect at 100 ms SOA, even though not significantly different from controls (*t*(11) = −0.793, *p* = 0.445; and *t*(11) = −1.767, *p* = 0.105, respectively). At 100 ms SOA, RE showed a normal cue-size effect, not significantly different from that of the controls (*t*(11) = 0.176, *p* = 0.313). At the 500 ms SOA, in contrast, AS (*t*(11) = 0.793, *p* = 0.445), and CG (*t*(11) = 0.870, *p* = 0.403) showed a cue-size effect even larger than controls, while RE showed an inverted cue-size effect (*t*(11) = −1.167 *p* = 0.368).

Therefore, the control groups showed an advantage in the detection of the target stimulus when it was preceded by the presence of a small-square cue, which enables the reduction of the visual space required to focus. In particular, according to the cue-size effect results, the effect of the focal component seemed stronger in the exogenous condition (i.e., short SOAs). More interestingly, all the three tested patients showed some difficulties in controlling the attentional focus, that is, inverted cue-size effects in one of the two SOA conditions. Nevertheless, the small group of controls did not allow us to find significant differences.

## 3. Experiment 2

With respect to Experiment 1, we then reversed the reasoning: it is not only true that those who make substitution errors should show an impairment of flexibility in modifying the magnitude of the focus of attention, but the opposite should also be true. Patients with difficulty in controlling focal attention should show substitution errors in reading aloud single visual verbal stimuli. Therefore, we started from the performance in the T detection task and compared, one by one, the performance of brain-damaged patients with that of a control group. We only used the presence of a right-focal lesion (to increase the likelihood of a peripheral reading deficit) and their willingness to participate in the research as the criteria for the selection of patients.

Once the patients with focal attention deficit were identified, we verified their ability to read words and pseudowords, spaced or not, with the aim of verifying the presence of substitution errors and the influence of crowding. Crowding is, indeed, greater with a reduced inter-letter space and decreases inversely [27,28,42].

### 3.1. Participants

#### 3.1.1. Control Participants

A total of 21 healthy participants (13M, mean age 68.9 ± 2.9 years, age range 65–73, mean education 10.0 ± 3.8 years, education range 5–16) took part in the study and served as control group. They were all right-handed and had no history of previous neurological and psychiatric disorders.

#### 3.1.2. Brain-Damaged Patients with Reading Difficulties

Seventeen right-brain-damaged patients (eight females) were enrolled in the study from the inpatient population of Casa di Cura Privata del Policlinico, Milan. Sixteen patients had suffered a stroke (3 ischemic, 10 haemorrhagic, 3 ischemic with haemorrhagic infarction), while one patient was hospitalized after parietal meningioma resection. The mean length of the disease was 19.4 months (SD ± 37.9; range 0.5–152.8). The mean age of the group was 66.24 years (SD ± 13.51; range 39–86) and mean education 10.12 years (SD ± 3.81; range 5–17). The patients were all right-handed and had no history of previous neurological and psychiatric disorders. The presence of unilateral spatial neglect was assessed by means of the following tasks: letter [43] and Bell cancellation [35], star cancellation [44], and apple cancellation [45,46]. Demographic and neurological data for the patients are summarized in Table 4. The project was approved by the local Ethical Committee, and informed consent was obtained from all participants, in accordance with the Declaration of Helsinki.

### 3.2. Materials and Methods

**Experiment on attentional focus.** The paradigm was the same as in Experiment 1 but with a few differences.

Stimulus presentation, timing, and response recording were carried out by the E-prime 2.0 software (Psychology Software Tools, Pittsburgh, PA, USA).

Moreover, the target stimulus appeared after one of three possible SOAs of 100, 300, or 500 ms.

The participants completed 160 trials. SOA conditions and cue types were presented randomly. At the beginning of the experiment, a practice session comprising 25 trials was run in order to allow the participants to familiarize themselves with the task and to practise with response modality.

**Letter by letter analysis of reading errors.** A list of 80 words and 80 pseudowords was used. The words were selected from the database produced in a previous study [38] and they varied in length, being five or seven letters long (40 stimuli for each length). The 80 pseudowords were created from these words by changing two or more letters in such a way that the stimuli obtained were very different from the original. The stimuli were printed in capital Courier New font, which is characterized by consistent letter spacing, and the size was kept constant (40 pt) and subtended 1.0°. Words and pseudowords were written in black and presented on a white background. Stimuli were presented in two conditions: standard font spacing (uniform spacing condition) and spaced (centre-to-centre letter spacing proportional to each letter’s eccentricity). Letter size was kept constant (1 deg, × letter height). Letter spacing varied by a factor of 2 from 1° for foveal letters to 7° for the most eccentric letter couples in the seven-letter word condition.

The participants were seated in front of a 17-inch CRT monitor. A chin and forehead rest stabilized their head position and kept the viewing distance constant at 57 cm. The eye movements of the participants were monitored by an SR Research EyeLink 1000 eye-tracker controlled by the SR Research Experiment Builder software (SR Research Ltd., Canada), in order to control for fixation accuracy.

In each trial, the patients were shown two small square fixation points, vertically displaced 1.5° apart on the centre of the screen. These fixation marks remained on the screen for the entire experimental session. Stimulus onset was triggered when the patient steadily fixated on the centre of the space between the two square marks for at least 50 ms. This procedure ensured that the first fixation landed on the centre of the stimulus. Each trial was presented at the centre of the screen between the fixation marks (i.e., the central letter of each stimulus was vertically aligned to the fixation marks) and remained on the screen until the onset of the patient’s response. There was no time constraint for responding, and the participants were asked to read aloud each stimulus as accurately as possible. Words and pseudowords appeared in random order across participants but were blocked for stimulus type. The responses were digitally recorded, and errors were subsequently scored after listening to the recorded track.

Errors were collapsed across conditions (spaced and unspaced) and expressed in percentages of omitted or substituted letters relative to the total number of letter positions presented.

### 3.3. Results

Reaction times (RTs) were adopted as the dependent measure. Atypical RT outliers (employing as a criterion 2.5 SDs above or below the mean within each participant) were discarded and not analysed. Controls’ data were analysed via two-way repeated-measures ANOVA, and the effects of interest were those associated with the experimental manipulations—that is, Cue (big square, small square, dot, and absence of cue), SOA (100, 300 and 500), and their mutual interactions. The effect size in the ANOVA was also measured by computing the Eta Squared (η2). The cue-size effect was also computed in order to obtain an amodal quantification of the advantage of narrowing the focal component.

The ANOVA on the control participants proved as significant effects both the main factors of SOA (F(2,40) = 12.95, *p* < 0.001, η2 = 0.393) and Cue (F(3,60) = 3.60, *p* = 0.018, η2 = 0.153), whereas their mutual interaction was not significant (F(6,120) = 1.80, *p* = 0.105, η2 = 0.082). RTs were faster in the 300 and 500 ms SOA condition (respectively 427 and 426 ms) compared to the 100 ms condition (459 ms), and when the cue consisted of the small square (418 ms) compared to the other conditions (big square: 449 ms; dot: 445 ms; absence of cue: 439 ms). A planned comparison on the main effect of Cue showed that participants responded significantly faster after the small square condition than all the other cues (all ps < 0.05).

In order to obtain a quantification of the advantage of narrowing the focal component, we calculated the overall cue-size effect for controls for each SOA condition (i.e., RT for the big cue condition—RT for the small cue condition). Finally, we compared each patient’s performance with the control group in terms of cue-size effect by following the procedure described in Crawford et al. [47].

**Figure 3:**
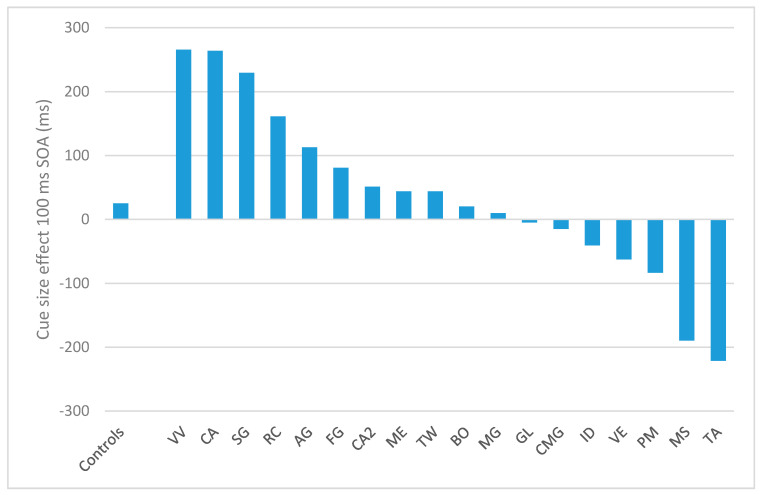
Results of the T detection task for the 17 patients (sorted by their cue-size effect) and the control groups (to the left), in terms of cue-size effect for the 100 ms SOA condition. Positive values indicate the presence of a cue size effect whereas negative values indicate the absence of such effect.

In the 100 ms SOA, the cue-size effect for controls amounted to 24.8 ms (d.s. = 61.4). Only four patients showed a significantly opposite performance as compared to controls: PM (−83.5; *t* = −1.7; *p* = 0.05), VE (−62.4; *t* = −1.4; *p* = 0.08), TA (−221.5; *t* = −3.9; *p* = 0.0004), and MS (−189.6; *t* = −3.4; *p* = 0.001), indicating that, differently from controls and the other patients, they did not benefit from the cue narrowing. The other patients showed either a not significantly different performance from controls (AG, BO, CA2, CMG, GL, ID, ME, MG) or a larger cue-size effect (CA, SG, VV, RC). Results are reported in Figure 3. In the 300 SOA condition, the cue-size effect for controls was 22 ms (d.s. = 102 ms). In this condition, only the patient TA had a significantly different performance as compared to controls (−315 ms; *t* = −3.2, *p* = 0.002). Two patients, CA2 and RC, had a significantly bigger cue-size effect as compared to controls (CA2: 222ms, *t* = 1.9, *p* = 0.03; RC: 332 ms, t = 2.97, *p* = 0.03). None of the other patients differ from controls (*p* > 0.05). For the 500 ms SOA condition, controls had a cue-size effect of 48 ms (sd = 91 ms). The following patients had a significantly different effect as compared to controls: AG (−240 ms, t = −3.1, *p* = 0.002); VV (−149 ms, t = −2.1; *p* = 0.02); PM (−387 ms, t = −4.7; *p* = 0.0001); and RC (−256 ms, t = −3.3, *p* = 0.002). None of the other patients differ from controls (*p* > 0.05).

## 4. Results

This section may be divided by subheadings. It should provide a concise and precise description of the experimental results, their interpretation, as well as the experimental conclusions that can be drawn.

The four patients significantly worse than controls in the 100 ms SOA condition of the T detection task were the only ones who made a larger number of substitutions compared to omissions (Table 5).

**Figure 4:**
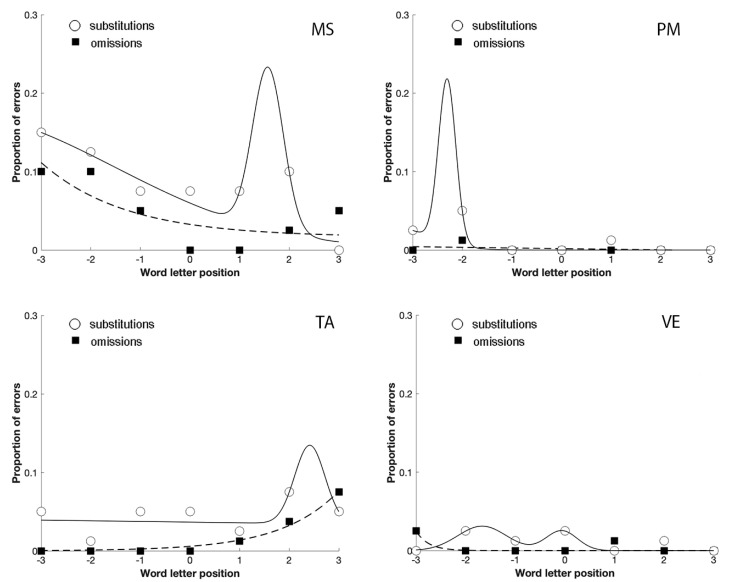
The distribution of omission and substitution errors made by the four patients with a prevalence of substitution errors in Experiment 2 in the unspaced condition. 0 corresponds to the central letter in the string, positive values to the letters on the right and negative values to the letters on the left.

**Figure 5:**
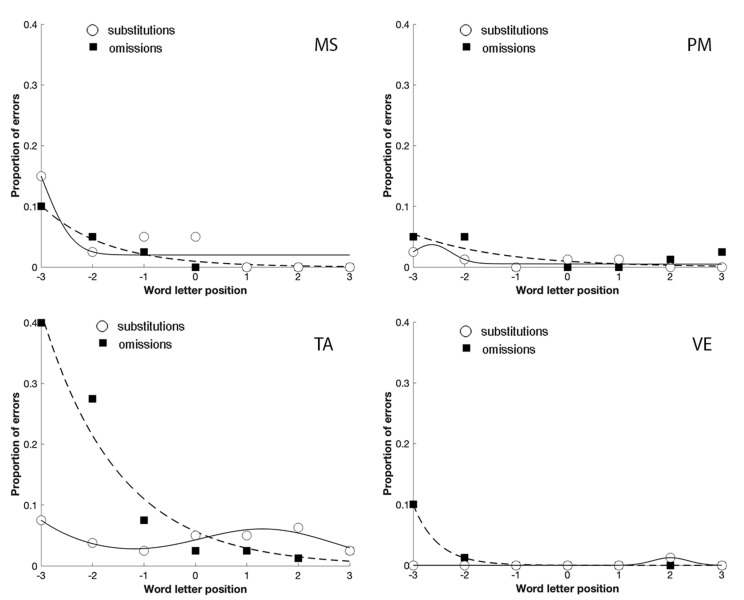
The distribution of omission and substitution errors made by the four patients with a prevalence of substitution errors in Experiment 2 in the spaced condition. 0 corresponds to the central letter in the string, positive values to the letters on the right and negative values to the letters on the left.

The analysis of their error distribution of substitutions and omissions, in the unspaced and spaced conditions, further confirmed the relationship between focal attention and substitution errors in reading (Figure 4; Figure 5). Indeed, in the case of omissions, the exponential decay captures a large proportion of variance for MS (unspaced R^2^ = 0.64; spaced R^2^ = 0.98), TA (unspaced R^2^ = 0.70; spaced R^2^ = 0.95), and VE (unspaced R^2^ = 0.73; spaced R^2^ = 1); PM made omission errors only in the spaced condition (R^2^ = 0.53), whilst substitution errors showed a bimodal distribution for MS (unspaced R^2^ = 0.96; spaced R^2^ = 0.83), PM (unspaced R^2^ = 0.93; spaced R^2^ = 0.65), TA (unspaced R^2^ = 0.50; spaced R^2^ = 0.88) and VE (unspaced R^2^ = 0.79; spaced R^2^ = 1).

The comparison between Figure 4; Figure 5 shows that the spaced condition was associated with a reduction of the substitution errors, as predicted by crowding. The opposite was observed with omission errors that increased with spacing, replicating the results obtained by Martelli et al. [22]. Finally, omissions were lateralized to the left-side of the stimulus, as predicted by USN. In contrast, substitutions were more equally distributed across the entire stimulus.

Therefore, the results of Experiment 2 confirmed that patients with a focal attention deficit were characterized by a substitution-type reading deficit as well, sensitive to space manipulation in the direction predicted by crowding.

## 5. Discussion

The suggestion that the prevalence of omission or substitution errors can disclose different deficits is not a new one (e.g., [22,23,25,48]). Previous studies have shown that a prevalence of omission errors is associated with neglect [22,23] and inaccurate fixations [24,26]. It represents the majority of neglect dyslexia patients and is easier to address in group studies.

Conversely, the prevalence of substitution errors is rare and mostly found in single-case studies [1,10,11,21,25]; patients who mostly substituted have often been described as having no other sign of neglect (see [17]).

In accordance with Martelli et al.’s [22] dual model, omission errors were lateralized on the left part of the stimulus, whereas substitution errors were more distributed across the entire stimulus, sparing the centre and the first and last letters of the word, as predicted by crowding. Furthermore, substitution errors, according to a perceptual integration mechanism, occur both in the presence and absence of USN, confirming their independence from it.

The present study was aimed at investigating the mechanism underlying substitution errors in acquired peripheral dyslexia. Previous studies have demonstrated that the attentional focus can be modified according to the task demands in foveal vision [39,49]. Moreover, Albonico et al. [34] showed that in healthy individuals the adjustment of the size of the attentional window in the fovea affects the crowding threshold. These results suggest that focal attention has a role in reducing crowding under normal conditions and that, in the case of deficit, it could enhance such a phenomenon.

Therefore, we have aimed to verify that a deficit of focal attention could determine a pathological crowding effect, leading to imprecise letter identification and, consequently, substitution errors.

In Experiment 1, the three patients characterized by a prevalence of substitution errors in reading single pseudowords were administered with a paradigm of focal attention, with the aim of verifying the hypothesis that the altered-features integration process that produces substitution errors is a consequence of the inability to control and adjust the size of the attentional focus. Our results provided further confirmation that the attentional focus can be modified in foveal vision under different cue characteristics and temporal constraints [33,39,49]. Indeed, the control participants showed an advantage in the detection of a target stimulus preceded by the presence of a small cue, which allowed the visual space required to focus to be reduced. Furthermore, this advantage was stronger when the SOA between the cue and the target stimulus was short (i.e., exogenous condition). In contrast, two out of the three patients characterized by substitution-type reading errors showed an impairment in the control of the attentional focus for short SOAs. Their cue-size effect was reversed. The third patient showed a reversed cue-size effect for a longer SOA.

Due to the small number of patients with a prevalence of substitution errors and with the aim of strengthening the relationship between focal attention deficit and substitution errors, in Experiment 2 we selected the patients on the basis of a T detection task and then observed their performance within the word and pseudoword reading task. We verified that the 4 out of 17 patients who significantly differed from healthy controls in performance at the focal attention paradigm were those who showed a prevalence of substitution errors in reading words and pseudowords.

Moreover, the letter by letter analysis or reading errors of the selected patients showed a bimodal distribution of substitution errors and an effect of spacing, suggesting an enhancement of the crowding phenomenon.

We suggest that the deficit in the control of attentional focus might determine the performance of the patients during the reading task and their substitution errors. In particular, in foveal vision, focusing on a smaller area of visual space can increase the spatial resolution and enhance the feature integration process up to the structural limit of visual acuity. A failure in the control of attentional focus (both endogenous and exogenous) would thus prevent the “isolation” of the letters from each other, and therefore it would not allow the patients to identify every single one correctly. Moreover, as further confirmed by the results of Experiment 3, this impairment is more evident in the normal reading condition, whereas when the inter-letter spacing is increased the deficit is less pronounced, because the overlapping of the integration fields of different letters is reduced in this latter case.

To summarize, in accordance with the dual model of reading type errors of Martelli et al. [22], we have investigated in the present study the specific mechanisms underlying substitution errors. In particular, we have demonstrated that patients affected by substitution-type reading errors show a deficit in controlling the attentional focus, and we suggest that this impairment is the underlying reason that causes the prevalence of substitution errors in acquired peripheral dyslexia. In turn, we have confirmed that patients with a prevalence of substitution errors show an abnormal feature integration process (i.e., crowding) that is reduced by increasing the inter-letter spacing. Overall, this study is novel in that it links the altered-features integration process that causes substitution errors to the impairment of the control of the focal component of selective attention.

## 6. Conclusions

As proposed by Manassi and Whitney [50], crowding, as a phenomenon, occurs at different levels of information processing, and this suggests that the mechanisms that reduce crowding and facilitate object recognition can be more than one.

Here we propose that focal attention allows the rapid adaptation of the field of integration to the proper size of the object for its feature integration and recognition; such a mechanism can decrease crowding in the fovea and parafovea (e.g., [34]) by reducing the integration window in the case of small objects such as letters. A compromised focal attention cannot help in reducing crowding and thus impairs reading.

According to the actual classification of acquired peripheral reading disorder, the patients with a deficit in reading words and pseudowords, mainly characterized by substitution errors, can be included within the group of peripheral dyslexias and, more specifically, within spatial dyslexia [51]. Nevertheless, this should be considered the sign of a deficit involving one of the visuospatial components of attention, namely focal attention.

## Figures and Tables

**Table 1:** Demographic and neurological features for the three patients selected. Lesions: F, frontal lobe; P, parietal lobe; T, temporal lobe; O, occipital lobe; C, cerebellum. Gender: M/F, male/female.

Sex/Age/Education	Duration of Disease (Months)	Etiology	Visual Half-Field Deficits	Lesion Site
RE M/74/10	22	Ischemic stroke	-	F-P-O + bg dx
AS M/78/12	2	Ischemic stroke	-	O dx + C sx
CG M/68/17	2	Ischemic stroke	-	P-T sx

**Table 2:** Baseline assessment of Unilateral Spatial Neglect (USN) and Neglect Dyslexia (ND) for the three patients.

	RE	AS	CG
**Neglect Assessment**			
Bells cancellation			
(omissions)			
Left (/27)	4	3	0
Right (/27)	0	4	0
Bisection ^1^ (mm)	-	0.0	13.6 *
**Reading Assessment**			
Words			
Errors	3/38	4/38	5/38
Neglect errors	2/3	3/4	3/5
Omissions	0/2	0/3	1/3
Substitutions	2/2	3/3	2/3
**Pseudowords**			
Errors	13/38	9/38	6/38
Neglect errors	10/13	8/9	5/6
Omissions	1/10	0/8	0/5
Substitutions	9/10	8/8	5/5

^1^ bisection task, 16-cm-lines bisection error in mm from the objective midpoint. * Pathological scores.

**Table 3:** Mean reaction times (and standard deviations) for controls and the three patients in the cueing task by SOA and Cue conditions.

RTs								
SOA 100					SOA 500			
	*none*	*big*	*small*	*dot*	*none*	*big*	*small*	*dot*
Controls	547 (126)	586 (144)	542 (115)	593 (130)	529 (124)	534 (132)	498 (118)	549 (148)
RE	490	544	531	535	487	451	494	505
AS	454	449	513	476	464	567	509	489
CG	561	495	527	532	502	593	613	549

**Table 4:** Demographic and neurological data of the 17 right-brain-damaged patients included in Experiment.2.

	Sex/Age/Education	Duration of Disease (Months)	Etiology/Lesion Site	Visual Half-Field Deficits	Letter Cancellation		Bell Cancellation		Star Cancellation		Apple Cancellation	
					*l*	*R*	*L*	*R*	*L*	*R*	*Egocentric score*	*Allocentric score*
CMG	F/51/13	3	IH/F P	ext	0/53	5/51	3/18	1/17	5/30 *	0/26	−1	0
CA	M/86/12	2	IH/Bg	-	2/53	4/51	1/18	2/17	0/30	1/26	−2	0
MS	M/83/13	2	H/Bg	na	50/53 *	44/51	16/18 *	10/17	8/30 *	0/26	17 *	2 *
RC	M/77/5	2	H/F T ins t Bg	+	53/53 *	26/51	18/18	13/17	23/30 *	5/26	7 *	4 *
VE	M/39/13	30	H/F T P Ins Bg	++	0/53	0/51	3/18	0/17	3/30 *	1/26	5 *	0
CA	M/57/17	1	H/t	ext	1/53	2/51	2/18	6/17	4/30	3/26	7 *	0
GL	F/63/13	1	H/t ic	-	0/53	0/51	1/18	2/17	2/30 *	0/26	1	0
VV	F/80/5	1	I/P	na	4/53	14/51	2/18	2/17	2/30	7/26	6 *	15 *
ID	M/63/13	20	H/F T P O ins t Bg	++	53/53 *	21/51	18/18 *	6/17	30/30 *	7/26	13 *	11 *
AG	M/70/5	54	H/F T P O ins	++	37/53 *	20/51	16/18 *	1/17	2/30 *	0/26	13 *	2 *
BO	M/55/8	38	H/t ic	-	0/53	0/51	0/18	1/17	0/30	0/26	0	0
TW	F/61/13	153	I/F P T ins	-	0/53	0/51	1/18	2/17	0/30	0/26	−6	0
MG	F/62/8	1	N/P	-	0/53	0/51	0/18	0/17	0/30	0/26	2	1
TA	F/79/5	1	IH/F T O	++	24/53 *	2/51	8/18	6/17	8/30 *	0/26	19 *	−1
ME	F/82/8	1	H/P O	++	9/53 *	0/51	4/18	1/17	10/30 *	2/26	0	50 *
SG	M/50/8	1	I/T P O	++	12/53 *	0/51	17/18 *	9/17	1/30	0/26	10 *	0
PM	F/68/13	19	H/F T P	ext	7/53 *	2/51	12/18 *	7/17	12/30 *	5/26	12 *	3 *

M/F: male/female; I/H/N: ischemic/hemorrhagic/neoplastic lesion. F: frontal; P: parietal; T: temporal; O: occipital; Ins: insula; ic: internal capsule; Bg: basal ganglia; t: thalamus. na, not available for mapping/not assessed. ext: extinction to double simultaneous stimulation. +/++, deficit; -, no deficit. L/R: left/right; * pathological performance according to the available normative data or control groups.

**Table 5:** Number of reading errors for the 17 patients in the standard spaced condition (unspaced). The patients who showed a prevalence of substitution errors in reading words and pseudo-words are highlighted in bold.

Patients	CMG	CA	MS	RC	VE	CA2	GL	FG	ID	AG	BO	TW	MG	TA	ME	SG	PM
Omissions	5	1	10	53	2	0	0	0	14	19	1	0	0	6	6	19	1
Substitutions	2	3	22	44	6	0	0	2	3	7	0	2	3	20	1	9	5

## Data Availability

The data presented in this study are available on request from the corresponding author. The data are not publicly available due to the consent provided by patients.
